# Selective Vulnerability of Neurons in Layer II of the Entorhinal Cortex during Aging and Alzheimer's Disease

**DOI:** 10.1155/2010/108190

**Published:** 2010-12-01

**Authors:** Alexis M. Stranahan, Mark P. Mattson

**Affiliations:** ^1^Department of Psychological and Brain Sciences, Johns Hopkins University, Ames Hall, 3400 N. Charles St., Baltimore, MD 21218, USA; ^2^Laboratory of Neurosciences, National Institute on Aging Intramural Research Program, Baltimore, MD 21224, USA

## Abstract

All neurons are not created equal. Certain cell populations in specific brain regions are more susceptible to age-related changes that initiate regional and system-level dysfunction. In this respect, neurons in layer II of the entorhinal cortex are selectively vulnerable in aging and Alzheimer's disease (AD). This paper will cover several hypotheses that attempt to account for age-related alterations among this cell population. We consider whether specific developmental, anatomical, or biochemical features of neurons in layer II of the entorhinal cortex contribute to their particular sensitivity to aging and AD. The entorhinal cortex is a functionally heterogeneous environment, and we will also review data suggesting that, within the entorhinal cortex, there is subregional specificity for molecular alterations that may initiate cognitive decline. Taken together, the existing data point to a regional cascade in which entorhinal cortical alterations directly contribute to downstream changes in its primary afferent region, the hippocampus.

## 1. Introduction

Both people and neurons, exhibit variability in terms of their vulnerability to cognitive decline. Just as some individuals remain functionally intact in their eighth decade of life, certain types of neurons maintain their capacity for plasticity over the lifespan. Conversely, others begin to show signs of cognitive decline at earlier ages, and in these individuals, specific brain regions exhibit molecular alterations that contribute to functional deficits by disrupting the local circuitry [1]. During normal aging, the loss of connectional integrity is not likely to be mediated by loss of neurons, as neuronal number is largely preserved [2]. Instead, subtle alterations in neuronal structure and biochemistry interact with changes in the cellular environment that are permissive for the onset of neuropathology [3].

The specific circuitry associated with age-related memory loss involves the synaptic relays within the medial temporal lobe system [4]. Sensory information converges on the entorhinal cortex, which then projects directly to the hippocampus via the perforant path. Within the entorhinal cortex, neurons in layer II are particularly susceptible to the deleterious consequences of aging, MCI, and AD [5], but the basis for their selective vulnerability remains unclear. Because the terminal zones of layer II neurons of the entorhinal cortex show the earliest evidence of regional atrophy in the context of age-related memory dysfunction [1], it is worthwhile to highlight certain aspects of their development, morphology, function, and molecular profile that may contribute to their precarious position in the aging brain. Layer II neurons are not homogeneous, and we outline possible mechanisms for selective vulnerability within this mixed population. Lastly, we explore potential mechanisms for transsynaptic spread, in which molecular alterations occurring within the layer II neurons of the entorhinal cortex influence cellular resilience and synaptic function within the hippocampus.

## 2. Development of Neurons in Layer II of the Entorhinal Cortex

The neural code underlying serial representation has been modeled using a “first in, first out” encoding principle [6]. Just as temporal order can determine the sequential activation of neuronal ensembles, it is possible that developmental ontogeny might contribute to differential trajectories during brain aging. During fetal development in rhesus monkeys, neurons in the entorhinal cortex are born earlier than neurons in other cortical regions and are also born before neurons that form the hippocampus [7]. There is some subregional heterogeneity with regard to entorhinal cortical development, such that in cats, the lateral entorhinal region develops before the medial subdivision [8]. However, the “first in, first out” hypothesis cannot account for the selective vulnerability of neurons in layer II, because if the earliest neurons born during development were also most susceptible to aging, then the deep neurons in layer V of entorhinal cortex would be most vulnerable, since the cortex forms in an inside-out pattern.

The chemoanatomical development of the entorhinal cortex also occurs earlier in development, relative to other cortical regions. Immunoreactivity for somatostatin and for the calcium-binding protein calbindin is detectable in the fetal rhesus monkey entorhinal cortex earlier than in other cortical regions [9]. While the developmental precocity of the entorhinal cortex may account for vulnerability of this region to the effects of aging, it does not explain the layer-specific susceptibility of layer II neurons.

## 3. Anatomy of Neurons in Layer II of the Entorhinal Cortex

Selectively vulnerable neurons share common characteristics across a variety of neurodegenerative diseases. Morphological complexity and the presence of long myelinated axons, likely contribute to neuronal vulnerability by increasing the amount of cellular machinery available to break down [10]. The increased surface area of morphologically complex cells might also increase susceptibility to toxic factors in the extracellular environment. Moreover, the bioenergetic requirements of neurons in the superficial layers of the entorhinal cortex are comparatively high [11], leaving them open to perturbation in aging. Total dendritic length in layer II neurons of the entorhinal cortex in macaques can reach 18.0 mm [12]. However, CA1 neurons in nonhuman primates exhibit similar dimensions in terms of their total dendritic length (~18.0 mm; [12]), and yet age-related cytoskeletal pathology occurs earlier in the entorhinal cortex than in area CA1 of the hippocampus [13]. Because the complexity of CA1 neurons is similar to that of entorhinal layer II neurons, selective vulnerability is unlikely to occur as a linear function of surface area.

**Figure 1:**
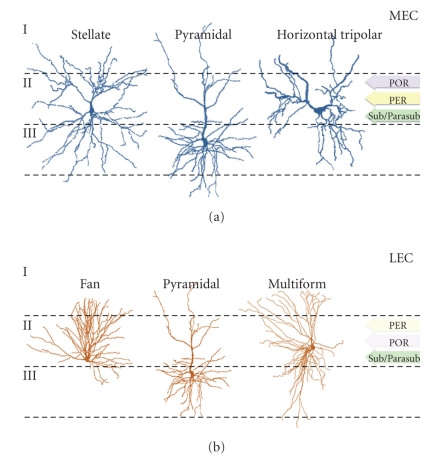
Morphological and connectional heterogeneity among principal neurons in layer II of the medial and lateral entorhinal areas. (a) In the medial entorhinal cortex (MEC), stellate cells are the most numerous class of excitatory neuron in layer II. However, pyramidal neurons and horizontal tripolar cells also reside in layer II and send afferents to the hippocampus via the perforant pathway. Layer II neurons in the MEC receive input from the postrhinal cortex (POR), and to a lesser extent from the perirhinal cortex (PER), and also from the subiculum/parasubiculum (Sub/Parasub) of the hippocampus. Whether these inputs preferentially contact a particular morphological class of layer II neuron remains to be determined. (b) In the lateral entorhinal cortex (LEC), fan cells are the most frequently observed, but pyramidal and multiform neurons also contribute axonal input to the perforant pathway. Layer II neurons of the LEC receive strong input from the PER, weaker input from the POR, as well as from the Sub/Parasub region of the hippocampus proper. All connectivity information is based on a review by Van Strien and colleagues [14].

Neurons in layer II of the entorhinal cortex are morphologically heterogeneous. Based on the framework established by Ramon y Cajal [15], Tahvildari and Alonso [16] divided the layer II neurons in the lateral entorhinal cortex of rats into three morphological subclasses: “fan” cells with dendrites extending horizontally out and up towards the pial surface; pyramidal cells with an apical dendrite ascending towards the pial surface and basal dendrites extending down into layer III; multiform cells, which did not fit into either category (Figure 1(b)). “Fan” cells were most numerous, and all three types of principal neurons were reported to exhibit dendritic spines. While no studies to date have identified molecular markers that distinguish between these classes of cells, their morphological differences likely contribute to differences in dendritic integration, firing patterns, and possibly, differences in their vulnerability to aging and AD.

In layer II of the medial entorhinal cortex, Klink and Alonso [17] again identified three anatomical cell types originally described by Ramon y Cajal [15]: stellate neurons with a short, thick apical dendrite that bifurcated within the borders of layer II; pyramidal neurons with a longer apical dendrite that bifurcated superficially in layer I; horizontal tripolar cells, with a horizontally oriented soma and dendrites that ascend diagonally towards the pial surface (Figure 1(a)). Stellate cells were more numerous, relative to pyramidal and horizontal tripolar cells. Again, dendritic spines were observed on all three classes of cells, suggesting that horizontal tripolar cells, despite their diagonal orientation, are not a type of interneuron. The anatomical diversity of neurons in layer II of the entorhinal cortex is likely to be associated with functional diversity, although no known molecular signatures exist for the distinct morphological subtypes. However, the variability in neuronal morphology in layer II might contribute to differences in susceptibility to aging and AD.

## 4. Electrophysiological Features of Neurons in Layer II of the Entorhinal Cortex

Just as the lateral and medial subdivisions differ in terms of neuronal morphology [16, 17], and in their developmental time of origin [8], the firing properties of neurons in these two areas differ in important ways. At the level of neuronal ensembles, the medial entorhinal cortex exhibits location-specific firing, while the lateral entorhinal cortex does not [18]. Within the medial entorhinal cortex, spatial selectivity exhibits a dorsal-ventral gradient, such that cells in the most dorsal components of the medial entorhinal cortex have smaller and more tightly spaced firing fields [19]. Neurons of the lateral entorhinal cortex fire in response to odor discrimination [20], including odor discrimination tasks that distinguish between conspecifics [21]. Differences in environmentally evoked firing likely arise from differences in afferent input to the medial and lateral entorhinal areas. The medial entorhinal cortex receives input from the postrhinal cortex, which primarily receives afferents from the visual and parietal cortices [22]. In contrast, the lateral entorhinal cortex receives direct olfactory input [23] in addition to receiving projections from other unimodal sensory regions via the perirhinal cortex [22] and subcortical input from the amygdala [22].

Combined lesions of the lateral entorhinal cortex and its primary afferent region, the perirhinal cortex, impairs contextual fear discrimination [24], opening the possibility that the perirhinal-lateral entorhinal flow of information may encode features of the environmental context [25]. Although this may prove to be the case, a definitive and unique role for lateral entorhinal neurons in some aspect of learning has not yet been elucidated, since combined lesions of the postrhinal and medial entorhinal cortices also reduced contextual discrimination in this study [24]. The available data suggest that the perirhinal-lateral entorhinal and postrhinal-medial entorhinal circuits serve complementary functions related to memory for contextual cues. 

Subregional and morphologically specific differences in the biophysical properties of neurons in layer II of the entorhinal cortex have also been reported. In layer II of the medial entorhinal cortex, stellate neurons exhibit subthreshold rhythmic oscillations, while pyramidal neurons did not [26]. The frequency of subthreshold oscillations [27] and integration of synaptic inputs [28] also covary with spatial selectivity along the dorsal-ventral gradient within the medial entorhinal cortex, suggesting that physiological mechanisms exist to account for grid-based firing in this region. In contrast, neither “fan” cells nor pyramidal cells in layer II of the lateral entorhinal cortex display subthreshold rhythmic oscillations [16], nor do they exhibit grid firing [18]. These differences in firing properties reinforce the idea that even within layer II of the entorhinal cortex, there may be distinct cellular mechanisms underlying differential susceptibility to aging and AD across the lateral and medial divisions.

## 5. Molecular Profile of Layer II Neurons and Their Niche in the Entorhinal Cortex

Several studies have used laser-capture microdissection and microarray techniques to identify gene expression signatures underlying the selective vulnerability of layer II neurons in the entorhinal cortex with aging and AD. One gene that is highly expressed in layer II entorhinal cortex is reelin [29], a large glycoprotein involved in neuronal development and synaptic plasticity [30]. It is fairly unusual for reelin to be found in excitatory neurons, as the majority of reelinergic cells in the hippocampus and in other cortical regions are interneurons [31]. However, a number of converging studies now suggest that reelin expression in the layer II neurons of the entorhinal cortex is associated with aging and neurodegenerative disease. 

Reelin is synthesized in the layer II neurons and transported along perforant path axons into the hippocampus proper [32]. Layer II neurons that are reelin-positive preferentially innervate granule neurons of the hippocampal dentate gyrus [33]. Reelin expression in layer II principal neurons of the entorhinal cortex is reduced in AD patient tissue and in animal models of AD [34]. This is not solely attributable to AD pathology, as naturally occurring variation in age-related cognitive decline correlates with the loss of reelin expression among layer II neurons in the lateral entorhinal cortex [3]. Because entorhinal cortical reelin expression fluctuates in concert with cognitive dysfunction, this specific change may initiate a cascade of signaling events leading to neuropathology in AD.

**Figure 2:**
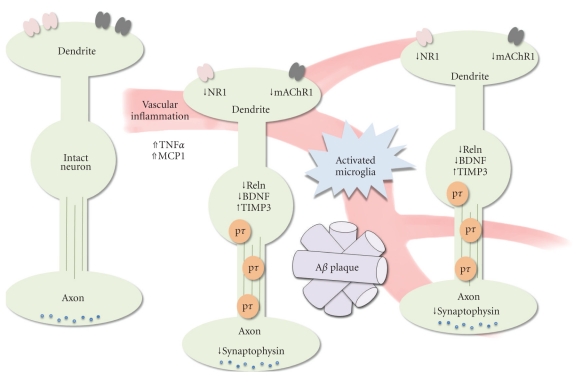
Microenvironmental changes interact with intrinsic cellular alterations to promote the selective vulnerability of entorhinal layer II neurons in aging and AD. Neurons located further away from plaques are likely to maintain a greater degree of structural and functional integrity in AD, while neurons situated close to plaques and in the vicinity of the vasculature are exposed to elevated levels of inflammatory cytokines such as tumor-necrosis factor-alpha (TNF alpha) and monocyte chemoattractant protein 1 (MCP1). Proinflammatory alterations in the local microenvironment, together with intrinsic changes in neuronal reelin (Reln), brain-derived neurotrophic factor (BDNF), and tissue inhibitor of metalloproteinase 3 (TIMP3) expression, could potentially impair synaptic function. This impairment would alter signal propagation both locally, through reductions in NMDA NR1 subunit and muscarinic acetylcholine receptor M1 (mAChR1) expression, and downstream in the hippocampus, through reductions in synaptophysin expression in the terminal fields for layer II entorhinal neurons.

Layer II neurons show a variety of molecular alterations in AD, including reductions in muscarinic acetylcholine receptor 1, GABAA receptor delta, and ionotropic glutamate receptor NMDA 1 (Figure 2; [35]. These changes occur in layer II neurons that do not exhibit histopathological tangles. In contrast, tangle-bearing neurons in layer II of the AD entorhinal cortex show increased expression of apolipoprotein-J and tissue inhibitor of metalloproteinase-3, relative to non-tanglebearing neurons [36]. The question of whether these molecular changes map onto the different morphological cell types that reside in layer II of the entorhinal cortex remains to be addressed. Additionally, tangle formation occurs earlier in the “transentorhinal region” [13], relative to the entorhinal cortex per se, and the molecular signatures that distinguish different areas within the entorhinal cortex have not yet been identified. Lastly, neurons located close to plaques show a variety of morphological and molecular alterations not observed in neurons located further away from plaques [37], but transcriptional alterations in plaque-neighboring neurons have not yet been characterized.

There are several features of the local environment of layer II entorhinal neurons that are altered in ways unfavorable for the continued function and survival of these neurons in aging and AD (Figure 2). Reduced neurotrophic support is one such alteration. There is a marked reduction in the amount of acidic fibroblast growth factor (aFGF) in the layer II niche in AD, and aFGF induces the expression of calbindin [38], a calcium-binding protein known to protect neurons against degeneration in experimental models relevant to the pathogenesis of AD [39, 40]. Moreover, levels of BDNF are reduced in the entorhinal cortex in AD [41], and viral vector-mediated expression BDNF ameliorated memory and synaptic LTP deficits in a rat dementia model in which synapse-specific lesions were induced by botulinum toxin [42]. A BDNF signaling deficit is a particularly attractive mechanism for the selective vulnerability of layer II entorhinal neurons in aging and AD, because several factors believed to increase the risk of AD (excessive energy intake, a sedentary lifestyle, and diabetes) also reduce BDNF levels in multiple brain regions including target neurons of layer II cortical neurons [43].

Finally, there is evidence supporting increased inflammation within the local environment of entorhinal layer II neurons in AD (Figure 2). The entorhinal cortex receives vascular input from both the posterior and middle cerebral arteries [44], which form a dense reticulated network around the verrucae entorhinalis [45]. The redundancy of vascular input to this region may contribute to its selective vulnerability to blood-borne inflammatory factors. In the triple transgenic mouse model of AD, there is a selective increase in microglial activation and elevated levels of the proinflammatory cytokines tumor necrosis factor-alpha and monocyte chemoattractant protein-1 [46]. Selectively vulnerable neurons also exhibit elevated levels of proteins in the classical complement cascade [47], an innate immune pathway implicated in neuronal death in multiple neurodegenerative disorders including AD [48]. 

More recently, evidence has accumulated implicating hyperactivation of one or more toll-like receptors (TLRs) in microglia and/or neurons in the dysfunction and degeneration of neurons in the entorhinal-hippocampal system [49, 50]. For example, activation of TLR4 contributes to the neurotoxic effects of amyloid beta-peptide [51]. On the other hand, activation of TLR4 can stimulate the uptake of amyloid beta-peptide by microglia, thereby reducing the accumulation of amyloid in the brain [52]. Whether targeting innate immune signaling pathways can protect layer II neurons in the entorhinal cortex against aging and AD remains to be determined.

## 6. Circuit Susceptibility with Aging and AD: Evidence for Transsynaptic Spread

Age-related cognitive deficits are associated with functional and molecular alterations along the perforant path projection from layer II of the entorhinal cortex to the hippocampal dentate gyrus and CA3 fields (Table 1). Specifically, aged humans with mild cognitive impairment show synaptic loss in hippocampal regions that receive afferent input from layer II of the entorhinal cortex [53]. Moreover, aged rats that are cognitively impaired exhibit reduced presynaptic marker immunoreactivity in the terminal zones for the layer II inputs [54]. This change appears to be initiated presynaptically, as no alterations in postsynaptic marker expression were observed in this circuitry with aging and cognitive impairment [55]. Functionally, perforant path axons show reduced response amplitude in aged rats [56], and the threshold for induction of long-term potentiation is increased [57]. These observations point to circuit-specific vulnerability of the layer II projection from the entorhinal cortex to the hippocampus, but the degree to which entorhinal cortical signaling alterations cause molecular adaptations within the hippocampus remains unclear. 

The perforant path projection to the hippocampus is also selectively vulnerable in mouse models that exhibit neuropathological alterations similar to those observed in AD (Table 1). The outer molecular layer of the dentate gyrus, which receives input from layer II of the lateral entorhinal cortex, exhibits early amyloid *β*-peptide accumulation in AD models [58]. Likewise, the outer molecular layer exhibits accelerated synaptic loss in AD models [59]. The perforant path projection to the dentate gyrus also shows selective deficits in synaptic plasticity in AD models [60] and in normal aging [61]. Overall, these data demonstrate that the perforant path projection arising from neurons in layer II of the entorhinal cortex is particularly susceptible to molecular and functional alterations with aging and AD. 

Recent data suggest that transsynaptic spread of tau pathology might be one potential mechanism whereby pathological alterations in the entorhinal cortex influence cytoskeletal adaptations within the hippocampus proper. Microinjections of hyperphosphorylated tau led to the formation of neuropil threads in the distal afferent regions of the injection site [62], suggesting that like prion disease, tau pathology might spread across synapses. However, unlike prion protein, hyperphosphorylated tau itself cannot cross synaptic terminals, suggesting that some transsynaptic signal exists to inform the postsynaptic cell about the presence of hyperphosphorylated tau in the presynaptic cell. The nature of this molecular signaling cascade remains to be determined.

## 7. Summary and Conclusion

Developmental, morphological, functional, and molecular features of layer II neurons in the entorhinal cortex interact to promote the early susceptibility of this cell type to aging and AD. Different morphological subtypes exist within layer II neurons, with distinct firing properties that may render certain classes of neurons more sensitive to age-related functional adaptations. The molecular phenotype of tangle-bearing neurons is different from that of non-tangle-bearing neurons, suggesting that the presence of tangles elicits transcriptional alterations within layer II neurons of the entorhinal cortex. These transcriptional alterations may lead to synaptic loss and the onset of tau pathology within the hippocampus. In this regard, entorhinal layer II neurons represent a vulnerable cell type with the potential to initiate downstream cascades leading to functional decrements among neurons of the hippocampus.

## Figures and Tables

**Table 1:** Selective vulnerability of synaptic connections between entorhinal layer II neurons and their targets in the hippocampus across different species during aging and in models relevant to Alzheimer's disease. In this table, “cognitive aging” refers to studies where correlations with memory deficits were observed in aged populations, while “aging” refers to studies comparing across different time points without behavioral assessment. PP: perforant path; DG: dentate gyrus; APP: amyloid precursor protein.

Species	Nature of deficit	Vulnerable synapses	Reference
Human	Cognitive aging	PP > DG/CA3	[1]
Monkey	Aging	PP > DG	[63]
Dog	Aging	PP > DG	[64]
Rat	Cognitive aging	PP > CA3	[54]
Mouse	Aging	PP > DG	[61]
Mouse	APP mutation (Tg2576)	PP > DG	[65]
Mouse	APP mutation (PDAPP)	PP > DG	[58]
